# Seroprevalence and Risk Factors Associated with Contagious Caprine Pleuropneumonia in Western Amhara, Northwest Ethiopia

**DOI:** 10.1155/2019/9878365

**Published:** 2019-07-03

**Authors:** Askale Abrhaley, Mebrat Ejo, Tsegaw Fentie

**Affiliations:** ^1^Department of Biomedical Sciences, College of Veterinary Medicine and Animal Sciences, University of Gondar, P.O. Box 196, Gondar, Ethiopia; ^2^Centre for Innovative Drug Development and Therapeutic Trials for Africa (CDT-Africa), College of Medicine & Health Sciences, Addis Ababa University, P.O. Box 9086, Addis Ababa, Ethiopia; ^3^Department of Veterinary Epidemiology and Public Health, College of Veterinary Medicine and Animal Sciences, University of Gondar, P.O. Box 196, Gondar, Ethiopia

## Abstract

Contagious caprine pleuropneumonia (CCPP) has been identified as a significant problem in goat production, especially in the arid and semiarid lowland areas of Ethiopia. Even though CCPP was reported in most of the goat rearing areas of the country, there is no adequate information on the disease in the Amhara Region. Cross-sectional study was conducted from November 2016 to April 2017 in the districts of Western Amhara to estimate the seroprevalence and identify the associated risk factors for occurrence of the CCPP. The risk factors considered included age, sex, agroclimate, and districts. A competitive enzyme-linked immunosorbent assay (cELISA) was carried out on a total of 400 goat sera samples, out of which 34 samples were found seropositive for specific antibodies against CCPP, with the overall seroprevalence of 8.5% (95% confidence interval (CI) =5.8, 11.2). Among the epidemiological factors considered, age and sex of the goats were not significantly associated with CCPP seroprevalence (p>0.05). However, the seropositivity was slightly higher in adults (9.9%) and female goats (9.0%) compared to young (6.3%) and male (7.5%) goats, respectively. The analysis of seroprevalence by district shows that the seroprevalence of CCPP in Metema (OR=14.34; 95%CI= 1.80, 114.09; p=0.012) and Fogera (OR=9.99; 95%CI= 1.10, 91.16; p= 0.041) was significantly higher compared to other study districts. Multivariable logistic regression analysis also identified the district as a risk factor for the occurrence of a high seroprevalence of CCPP. The present study revealed the seroprevalence and the distribution of CCPP in Western Amhara districts, and hence appropriate control measures including regular investigation and vaccination should be implemented to alleviate the problem.

## 1. Introduction

Goats are amongst the chief economically essential livestock sector in Ethiopia, and they are vital sources of cash income, meat, and milk for smallholder farmers in various agroecological areas of the country [1]. Even though goats represent an abundant national resource of the country, their productivity is lowered by numerous factors, including feed shortage, low genetic potential, and infectious diseases [2–4]. Among infectious diseases, contagious caprine pleuropneumonia (CCPP), caused by* Mycoplasma capricolum* subspecies (subsp.) capripneumoniae (Mccp), is one of the major constraints of goat production [5]. This disease is highly contagious and characterized by severe fibrinous pleuropneumonia with high morbidity and mortality [6, 7]. According to the World Organization for Animal Health [8], CCPP is reported as prevalent in more than 30 countries; predominantly in Africa, the Middle East, and West Asia, which contained more than half of the world's goat population. In African countries, including Ethiopia, the disease is observed since 1980s and considered as OIE notifiable diseases [9, 10]. It has a high economic relevance within the context of goat farming as it directly affects goat productivity and also influences international trade of goats and goat products [5, 11]. The lack of appropriate facilities is a major constraint for the diagnosis of Mccp from prevalent CCPP cases in the goat population, as this disease is not easily differentiated from other unobserved mycoplasmas infections, and also it is difficult to evaluate the prevalence and its influence globally [9, 10]. Yet, the occurrence and distribution of the disease are unknown and it might be more widespread than the countries where Mccp has been identified and characterized [12–14].

In Ethiopia, various studies in different localities have reported the prevalence of CCPP; it ranges from 10% to 43% in different regional states of the country [15–18]. These studies showed that factors such as insufficiency of accurate diagnostic services, shortage of vaccination against CCPP, poor management, weather conditions, and concurrent infections contribute to a widespread occurrence of the disease in rural and different agroecological areas of the country [19]. As reported by African Union's Inter-African Bureau of Animal Resources, Ethiopia is among the countries consistently reporting the disease [20, 21]; the occurrence of the disease follows the introduction of an infected animal into a group of susceptible goats [5], and the disease is usually complicated along with other microorganisms, such as* Mannheimia haemolytica* and* Pasteurella multocida* [11, 19, 22]. In different areas of the country, the CCPP outbreak has been documented [20, 21, 23]; however, the epidemiological surveillance of the disease has not yet been well recognized. Even though CCPP was reported in some of the goat rearing areas of Ethiopia [16, 24, 25], there is no adequate information about CCPP in the Amhara region. Therefore; this study was planned to study the seroepidemiological status of CCPP and associated risk factors in Western Amhara districts.

## 2. Materials and Methods

### 2.1. Study Location

**Figure 1:**
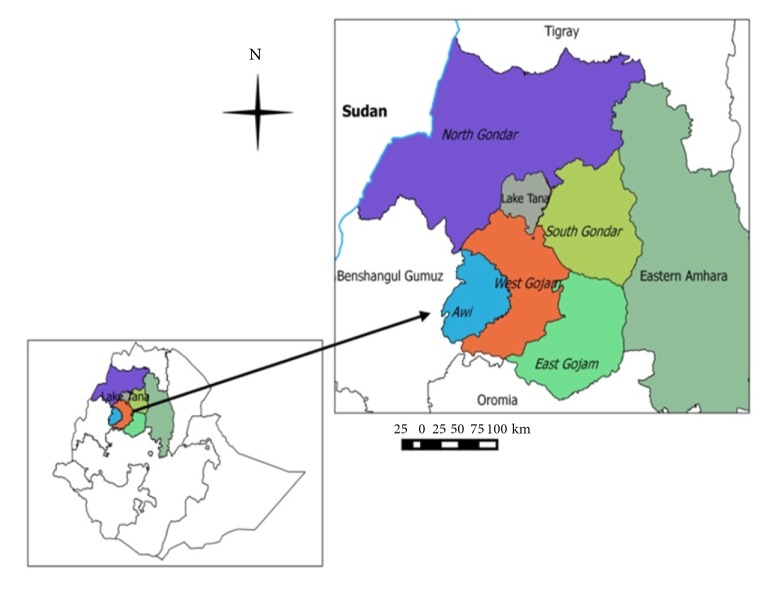
Map of the study area.

The study was conducted in selected zones of Western Amhara subregion, namely, Awi, West Gojam, East Gojam, and South and North Gondar (Figure 1), Northwest Ethiopia. The subregion is situated between 10.00-14.00° north latitude and 35.10-38.35° east longitude with the total annual rainfall ranging from 878 mm to 2100 mm and the annual average maximum and minimum temperature of 30.7°C and 22°C, respectively. Western Amhara subregion is characterized by different agroclimates with subsistence crop-livestock production [26] and ranked first in its small ruminant population in the Amhara regional state, Ethiopia. In the subregion, a number of people are involved in the agricultural system, including smallholder farming supplying food animals and animal products to the communities and the slaughterhouses. The subregion is surrounded by Benshangul Gumuz, Oromia, and Tigray regional states of Ethiopia and border cities of Sudan, with transboundary livestock movement among the nearby geographic areas.

### 2.2. Study Design and Sampling

A cross-sectional study was conducted between November 2016 and July 2017 to assess the seroprevalence and associated risk factors of CCPP in districts of Western Amhara subregion. A combination of multistage and systematic random sampling was used. Multistage sampling technique was applied for selecting the study districts and villages. Depending on the number of goat population and the agroecology of the area, a total of 10 districts (one from Awi zone, two from West Gojam, one from East Gojam, four from North Gondar, and two from South Gondar) and three villages per district were selected for this study. Systematic random sampling techniques were used to select participant households and study goats for blood sample collection. Householders owning goats and individual goat were primary sampling units for generating data and samples.

### 2.3. Study Animals

A total of 400 goats kept under traditional extensive farming system were included based on the formula given by Thrusfield [27] with 95% confidence level, 5% desired absolute precision, and considering 50% expected prevalence. Goats considered for sampling were those with no history of vaccination against CCPP, greater than 6 months old, and both sex groups. Age of study goats was determined based on owner's information and dental eruption. The goats were classified as young and adult, i.e., young if 1-1.5 years old and having up to four permanent teeth and adult if >1.5 years and having greater than four permanent teeth.

### 2.4. Serum Sample Collection

Five milliliter (5ml) of blood samples was collected from the jugular vein of each study goat in sterile nonheparinized Vacutainer. Collected blood samples were kept at room temperature in slant position for 1-2 hours and allowed to clot overnight at +4°C for serum separation. The separated serum samples were harvested into 5 ml sterile cryovial tubes and stored at −20°C in the Laboratory of College of Veterinary Medicine and Animal Sciences, University of Gondar, until serological analysis. Corresponding to each sample, the age, sex, and georeference information was collected and recorded on a separate predesigned recording sheet.

### 2.5. Serological Test

Competitive enzyme-linked immunosorbent assay (C-ELISA) [28] recommended by the International Organization for Animal Health [8] and CIRAD-UMR15 (France) was carried out for detection of CCPP. The assay was conducted by using the C-ELISA test kit (CIRAD-Montpellier, France) containing a monoclonal anti-Mccp antibody named MAb 4.52, precoated plates, and ready-made reagents. The assay was accomplished following the manufacturer's instructions. Briefly, serum samples to be tested were diluted and mixed with a specific monoclonal anti-*Mccp* antibody (Mab 4.52) in a preplate (uncoated plate) and then the homogenized contents of the preplate were transferred into the* Mccp* antigen coated microplates and incubated for one hour at 37°C with a gentle agitation. All of the wells were washed two times with washing solution. Anti-mouse IgG horseradish peroxidase conjugate was added to each well, and the plates were incubated for 30 min at 37°C. Following three times of washing, substrate solution 3,3′,5,5′-tetramethylbenzidine (TMB) was added to each well and incubated for 20 min at 37°C in a dark place. Finally, stop solution was added into each well with a gentle agitation allowing a color reaction to develop, and then the optical density (OD) of individual reaction was measured at 450 nm with an ELISA plate reader. Results were interpreted as per the manufacturer's instruction using the following formula: percentage of inhibition (PI)= ((OD Mab - test serum)/(OD Mab – OD conjugate))×100. That is, those sera with the PI greater than or equal to 55% were considered as positive for the presence of Mccp infection, and those sera with PI less than 55% were negative.

### 2.6. Statistical Analysis

The collected data and serology results were recorded in a Microsoft Office Excel spreadsheet. Statistical analyses were performed using STATA version 12 statistical software package (StataCorp, USA). Descriptive statistics such as frequency, percentage, or proportion were used to calculate the collected data. The overall seroprevalence was measured by dividing the number of seropositive goats by the total number of goats tested. Odds ratios (OR) and 95% confidence levels (CIs) were computed to measure the association of the seroprevalence of CCPP and the potential risk factors using binary logistic regression. The univariable and multivariable logistic regression were used to determine the association between risk factors and the serological status of goats. In the analyses, a confidence interval of 95% and a* p*-value of <0.05 were applied to determine statistical significance.

## 3. Results

### 3.1. Seroprevalence

A total of 400 sera samples of goats were included in this analysis. Of these, 43 sera samples were collected from Awi, 59 from West Gojam, 37 from East Gojam, 68 from south Gondar, and 193 from North Gondar zones. Among the total 400 sera samples tested by C-ELISA, 34 samples were found seropositive for specific antibodies against CCPP, Table 1, with the overall seroprevalence of 8.5% (95% CI=5.8, 11.2). Among the districts, the highest seropositivity for CCPP was detected in Metema district of the North Gondar zone (25.5%) and followed by Fogera district of South Gondar zone (19.2%), while lower seropositivity for CCPP was observed in Guangua district of Awi zone (2.3%). However, serum samples from Mecha district of West Gojam zone were found to be seronegative for CCPP, Table 1.

Among the zones included for sera sample collection, CCPP seroprevalence was higher in North Gondar zone (10.9%) followed by South Gondar (10.3%), East Gojjam (8.1%), and West Gojjam (3.4%), respectively. The least prevalence was reported in Awi zone (2.3%).

### 3.2. Association of Risk Factors and CCPP Seroprevalence

The univariable logistic regression analysis was computed to identify the effects of host and environment-related factors on the seroprevalence of CCPP, Table 2. The results of the univariate analysis showed that host-related factors such as age and sex of the goats were not significantly associated with CCPP seroprevalence (p>0.05). However, the seropositivity was slightly higher in adults (9.9%) and female goats (9.0%) compared to young (6.3%) and male goats (7.5%), respectively. In addition, the odds ratios (OR) of seropositivity in adult and female goats were 1.63 (95%CI= 0.76, 3.51; p= 0.212) and 1.23 (95%CI= 0.57, 2.65; p=0.598) times higher than young and male goats, respectively.

The environment-related risk factors, agroclimates and districts, were also analyzed by the univariable logistic regression. The seroprevalence was not significantly associated with the agroclimates (OR= 1.01; 95%CI= 0.46, 2.25; p> 0.05). The analysis of seroprevalence in districts shows that the seroprevalence of CCPP in Metema (OR=14.34; 95%CI= 1.80, 114.09; p= 0.012) and Fogera (OR=9.99; 95%CI= 1.10, 91.16; p= 0.041) was significantly higher as compared to other study districts, Table 2.

Among host and environmental-related factors, age and district were significant in the univariable analysis and were fitted to the final multivariable logistic regression model (data not shown). As a result, only the district was identified as a risk factor for the occurrence of a high seroprevalence of CCPP, Table 3.

## 4. Discussion

Contagious caprine pleuropneumonia (CCPP) is a disease of high economic relevance within the context of goat farming as it directly affects the productivity, most importantly, in the rural goat producing areas of developing countries [8, 23], including Ethiopia. Various studies in different settings have reported the prevalence of CCPP with varying results [23]. In the present study, the seroprevalence and associated risk factors of CCPP were assessed. The study revealed an overall seroprevalence of 8.5% (34 out of 400) in the studied goat population with widespread distribution and varied seropositivity among the study zones and districts. The seroprevalence of CCPP in this study was lower than that in some zones reported by Mekuria and Asmare [29] and Bekele et al. [30] in Ethiopia, Atim et al. [31] in Uganda, and Ingle et al. [32] and Hussain et al. [33] in Pakistan. In contrast, the seroprevalence finding in this study was higher than that reported by the same authors in other zones of Ethiopia [30] and in Uganda [31], Pakistan [32], and Turkey [34]. The variation in seroprevalence reports might be due to the differences in study areas, agroecological systems, goat management and production systems, population density, and the techniques used to define the seropositivity. In this study area where the traditional and extensive farming is practiced, CCPP might spread when goats meet at communal grazing and watering areas. As stated by Thiaucourt et al. [10], close contact between diseased and healthy animals is critical for the spread of CCPP to take place[10], and overcrowding and confinement have also been known to favor close contact and circulation of the disease in goat population. Stress factors such as malnutrition and movement over long distances have been also documented to enhance the spread and morbidity of the disease [29, 35]. Regassa et al. [15] and Sherif et al. [36] described that seroprevalence may also vary from one area to another within a country.

In the present study, there was a difference in the distribution of CCPP seroprevalence in the districts. A significantly higher seroprevalence of 25.5% (95% CI = 1.80, 114.09; p=0.012) and 19.2% (95% CI = 1.10, 91.16; p=0.041) was documented in Metema and Fogera districts, respectively. These findings were consistent with the results recorded by different research works conducted in different areas of Ethiopia [15, 16, 37]. Similarly, higher district level seroprevalences were also reported in Hammer and Benna-Tsemay districts of Southern Ethiopia [37] and Agago and Otuke districts of Uganda [31] with insignificant variation in the occurrence of CCPP. The difference in seroprevalence by districts could be explained by the differences in animal management system with common browsing and watering practices, the frequent animal movement to border regions, and the introduction of new animals. The study districts share boundaries with Benshangul Gumuz region of Ethiopia and Gallabat zones of Sudan; both of these areas practice extensive production system and common animal market route, which increases the spread of CCPP as the disease is a transboundary disease and highly contagious, transmitted by movement of infected goats [5, 8, 38, 39]. As reported by Kipronoh et al. [40], CCPP might also be predominantly prevalent in the neighboring areas with common borders and animal markets. Similar suggestions were also given by different studies conducted in Ethiopia [16, 24, 41].

In our study, host level variables such as age and sex of goats were not significantly associated with CCPP seropositivity. The study recorded slightly higher seropositivity in adults and female goats compared to young and male goats, respectively. In addition, the odds ratio (OR) of seropositivity revealed that adult and female goats were showing antibodies against CCPP more than young and male goats, respectively. This finding agrees with the reports by Hadush et al. [16] and Kipronoh et al. [42]. Both age groups and sexes of goats have been reported to be susceptible although higher mortalities have been documented among young animals than adults [29, 43]. This might be explained by the fact that adult goats have been at risk of exposure for a longer period than young animals and not necessarily as a result of new infection. It might also be due to reduced level of host protection mechanism and poor physiological condition in adult and female goats. Furthermore, goats may be exposed to adverse weather condition and malnutrition during their lifetime, which predisposes them to CCPP. It has also been reported that CCPP is highly contagious and fatal to susceptible goats irrespective of age and sex [19, 44]. Though all age groups are susceptible and seropositivity may be high in adult goats but mortality is higher in young animals than in adults [7, 11, 45, 46], and thus acutely infected young animals may die of CCPP before developing antibodies and not be available for testing [30, 42, 47].

Although our study presented an insignificant association (p > 0.05) between agroclimates and seropositivity, slightly higher CCPP seroprevalence was recorded in both lowland and midland areas. This finding is consistent with the study conducted in Eastern Ethiopia [48]. This might be linked to the movement of animals among the areas where the disease is highly prevalent for marketing, watering, and grazing, which increase the contact between animals and the spread of the disease. Additionally, goats in these agroclimates are more confined to browsing and watering areas, which might easily transmit CCPP to susceptible animals. Environmental stress, particularly hot and humid climate, favors precipitation of this disease [32]. Moreover, taking of goats to feeding and watering areas during dry season increases the contact of infected and susceptible goats as well as transmission of CCPP [40].

## 5. Conclusion

This study revealed that CCPP is among the most prevalent and widespread diseases of goats in the study area. Variation in the seroprevalence and distribution of the disease were observed among the study districts with high magnitude in Metema and Fogera districts. Therefore, control measures by regular surveillance and vaccination against CCPP should be implemented to mitigate the problem, including proper management of goat especially during outbreaks and awareness creation among the farmers about the means of prevention and control of the disease.

## Figures and Tables

**Table 1:** Seroprevalence of contagious caprine pleuropneumonia (CCPP) by study districts in Western Amhara subregion, Northwest Ethiopia.

Zone	District	Number of sera tested	Number of positives	Prevalence (%)
Awi	Guangua	43	1	2.3

West Gojam	Mecha	26	0	0
	Jabi-Tehnan	33	2	6.1

East Gojam	Debre Elias	37	3	8.1

South Gondar	Fogera	26	5	19.2
	Simada	42	2	4.8

North Gondar	Lay Armachiho	67	3	4.5
	Metema	55	14	25.5
	West Belessa	21	1	4.8
	Gondar Zuria	50	3	6.0

**Table 2:** Univariable analysis of risk factors for CCPP seropositivity classified by host- and environment-related factors.

Variables		Tested samples	Prevalence (%)	Odds Ratio (OR)	95% Confidence interval (CI)	p-value
Age	Young	158	6.3	Ref	-	-
	Adult	242	9.9	1.63	0.76, 3.51	0.212
Sex	Male	134	7.5	Ref	-	-
	Female	266	9.0	1.23	0.57, 2.65	0.598
Agro-climate	Lowland	295	8.5	Ref	-	-
	Midland	105	8.6	1.01	0.46, 2.25	0.976
District	Guangua	43	2.3	Ref	-	-
	Lay-Armachiho	67	4.5	1.97	0.20, 19.57	0.563
	Metema	55	25.5	14.34	1.80, 114.09	0.012
	Gondar Zuria	50	6.0	2.68	0.27, 26.77	0.401
	WestBelesa	21	4.8	2.10	0.12, 35.32	0.606
	Fogera	26	19.2	9.99	1.10, 91.16	0.041
	Simada	42	4.8	2.10	0.18, 24.07	0.551
	Mecha	26	0	-	-	-
	Jabi-Tehnan	33	6.1	2.71	0.24, 31.24	0.424
	Debre-Elias	37	8.1	3.71	0.37, 37.25	0.266

CCPP= contagious caprine pleuropneumonia; CI= confidence interval; OR= odds ratio; Ref= reference.

**Table 3:** Multivariable logistic regression analysis of risk factors of CCPP seropositivity by host- and environment-related risk factors.

Variables	Odds Ratio (OR)	95% Confidence interval (CI)	P-value
Age	1.61	0.74, 3.46	0.231
District	0.89	0.80, 0.99	0.045

CCPP= contagious caprine leuropneumonia; CI= confidence interval; OR= odds ratio.

## Data Availability

The data sets used and/or analysed during the current study are available from the corresponding author on reasonable request.

## References

[1] Yami A., Markel R. C. (2008). Sheep and Goat Production Handbook for Ethiopia. *Ethiopia Sheep and Goat productivity Improvement Program*.

[2] Gizaw S., Abegaz S., Rischkowsky B., Haile A., Okeyo A. M., Dessie T. (2013). *Review of sheep research and development projects in Ethiopia*.

[3] Ahuya C., Okeyo A., Mwangi-Njuru A. M., Peacock C. (2005). Developmental challenges and opportunities in the goat industry: The Kenyan experience. *Small Ruminant Research*.

[4] Boyazoglu J., Hatziminaoglou I., Morand-Fehr P. (2005). The role of the goat in society: past, present and perspectives for the future. *Small Ruminant Research*.

[5] Nicholas R., Churchward C. (2012). Contagious caprine pleuropneumonia: new aspects of an old disease. *Transboundary and Emerging Diseases*.

[6] Thiaucourt F., Bolske G. (1996). Contagious caprine pleuropneumonia and other pulmonary mycoplasmoses of sheep and goats. *Revue Scientifique et Technique de l'OIE*.

[7] Radostits O. M., Gay C. C., Hinchcliff K. W., Constable P. D. (2007). *Veterinary medicine: A Text Book of the diseases of Cattle, Horses, Sheep, Pigs and Goats*.

[8] OIE Terrestrial Manual. Contagious Caprine Pleuropneumonia Aetiology Epidemiology Diagnosis Prevention and Control References. Oie. 2012

[9] Lefevre P. C., Blancou J., Dedieu L., Diallo A., Libeau G., Thiaucourt F. (1993). Field diagnostic kits: a solution for developing countries?. *Rev Sci Tech*.

[10] Thiaucourt F., Bolske G., LENEGUERSH B., Smith D., Wesonga H. (1996). Diagnosis and control of contagious caprine pleuropneumonia. *Revue Scientifique et Technique de l'OIE*.

[11] Thiaucourt F., Bölske G. (1996). Contagious caprine pleuropneumonia and other pulmonary mycoplasmoses of sheep and goats. *Revue Scientifique et Technique de l'OIE*.

[12] Lorenzon S., Wesonga H., Ygesu L. (2002). Genetic evolution of Mycoplasma capricolum subsp. capripneumoniae strains and molecular epidemiology of contagious caprine pleuropneumonia by sequencing of locus H2. *Veterinary Microbiology*.

[13] Woubit S., Lorenzon S., Peyraud A., Manso-Silván L., Thiaucourt F. (2004). A specific PCR for the identification of Mycoplasma capricolum subsp. capripneumoniae, the causative agent of contagious caprine pleuropneumonia (CCPP). *Veterinary Microbiology*.

[14] Woubit S., Manso-Silván L., Lorenzon S. (2007). A PCR for the detection of mycoplasmas belonging to the Mycoplasma mycoides cluster: Application to the diagnosis of contagious agalactia. *Molecular and Cellular Probes*.

[15] Regassa F., Netsere M., Tsertse T. (2010). Sero-prevalence of contagious caprine pleuropneumonia in goat at selected woredas of Afar region. *Ethiopian Veterinary Journal*.

[16] Hadush B., Eshetu L., Mengistu W., Hailesilassie M. (2009). Seroprevalence of contagious caprine pleuropneumonia in Kefta Humera, Alamata (Tigray) and Aba-'ala (Afar), Northern Ethiopia. *Tropical Animal Health and Production*.

[17] Sharew A., Staak C., Thiaucourt F., Roger F. (2005). A Serological Investigation into Contagious Caprine Pleuropneumonia (CCPP) in Ethiopia. *Tropical Animal Health and Production*.

[18] Gizaw D., Gebreegziabher B., Ayelet G., Asmare K. (2009). Investigation of mycoplasma infection in goats slaughtered at ELFORA export abattoir, Ethiopia. *Ethiopian Veterinary Journal*.

[19] World OIE. (2008). Organization for Animal Health. Contagious caprine pleuropneumonia. *OIE Terr Man*.

[20] Interafrican Bureau for Animal Resources (2013). *Impact of Livestock Diseases in Africa*.

[21] Pan African Animal Resources (2013). *Pan African Animal Resources Yearbook*.

[22] Radostits O. M., Gay C., Hinchcliff K. W., Constable P. D. (2007). Disease caused by mycoplasma. *Veterinary Medicine. Textbook of the diseases of cattle, sheep, pigs, goats and horses*.

[23] Asmare K., Abayneh T., Mekuria S. (2016). A meta-analysis of contagious caprine pleuropneumonia (CCPP) in Ethiopia. *Acta Tropica*.

[24] Eshetu L., Yigezu L., Asfaw Y. (2007). A study on Contagious Caprine Pleuropneumonia (CCPP) in goats at an export oriented abattoir, Debrezeit, Ethiopia. *Tropical Animal Health and Production*.

[25] Gelagay A., Teshale S., Amsalu W., Esayas G. (2007). Prevalence of contagious caprine pleuropneumonia in the Borana pastoral areas of Ethiopia. *Small Ruminant Research*.

[26] Adugna A. (2014). *Amhara Demography and Health*.

[27] Thrusfield M. (2005). *Veterinary Epidemiology*.

[28] Thiaucourt F., Bölske G., Libeau G., Le Goff C., Lefèvre P. (1994). The use of monoclonal antibodies in the diagnosis of contagious caprine pleuropneumonia (CCPP). *Veterinary Microbiology*.

[29] Mekuria S., Asmare K. (2010). Cross-sectional study on Contagious Caprine Pleuro Pneumonia in selected districts of sedentary and pastoral production systems in Southern Ethiopia. *Tropical Animal Health and Production*.

[30] Bekele T., Asfaw Y., Gebre-Egziabeher B., Abebe G. (2011). Seroprevalence of contagious caprine pleuropneumonia in Borana and Guji lowlands, Southern Ethiopia. *Ethiopian Veterinary Journal*.

[31] Atim S. A., Ayebazibwe C., Mwiine F. N., Erume J., Tweyongyere R. (2016). A Survey for contagious caprine pleuropneumonia in Agago and Otuke districts in Northern Uganda. *Open Journal of Veterinary Medicine*.

[32] Ingle V. C., Sivakumar P., Kalorey D. R. (2008). Seroprevalence of contagious caprine pleuropneumonia in goats in Nagpur district of Vidarbha region. *Veterinary World*.

[33] Hussain R., Auon M., Khan A., Khan M. Z., Mahmood F., Ur-Rehman S. (2012). Contagious caprine pleuropneumonia in Beetal goats. *Tropical Animal Health and Production*.

[34] Cetinkaya B., Kalin R., Karahan M. (2009). Detection of contagious caprine pleuropneumonia in East Turkey. *Revue Scientifique et Technique de l'OIE*.

[35] Thiaucourt F., Guerin C., Mady V., Lefevre P. C. (1992). Diagnosis of caprine contagious pleuropneumonia: recent improvements. *Rev Sci Tech*.

[36] Sherif M., Addis M., Tefera M. (2012). Contagious Caprine Pleuropneumonia: Serological Survey in Selected Districts of Jijiga Zone, Ethiopia. *Asian Journal of Animal Sciences*.

[37] Mekuria S., Zerihun A., Gebre-Egziabher B., Tibbo M. (2008). Participatory investigation of Contagious Caprine Pleuropneumonia (CCPP) in goats in the Hammer and Benna-Tsemay districts of southern Ethiopia. *Tropical Animal Health and Production*.

[38] Awan M. A., Abbas F., Yasinzai M. (2009). Prevalence of Mycoplasma capricolum subspecies capricolum and Mycoplasma putrefaciens in goats in Pishin district of Balochistan. *Pakistan Veterinary Journal*.

[39] Awan M. A., Abbas F., Yasinzai M. (2010). First report on the molecular prevalence of Mycoplasma capricolum subspecies capripneumoniae (Mccp) in goats the cause of contagious caprine pleuropneumonia (CCPP) in Balochistan province of Pakistan. *Molecular Biology Reports*.

[40] Kipronoh K., Ombui J., Binepal Y. (2016). Risk factors associated with contagious caprine pleuro-pneumonia in goats in pastoral areas in the Rift Valley region of Kenya. *Preventive Veterinary Medicine*.

[41] Abera B. H., Eshetu L., Mengistu W. (July 2011). Seroprevalence of contagious caprine pleuropneumonia in Tigray and Afar, Northern Ethiopia. *Animal hygiene and sustainable livestock production. Proceedings of the XVth International Congress of the International Society for Animal Hygiene*.

[42] Kipronoh A. K., Ombui J. N., Kiara H. K., Binepal Y. S., Gitonga E., Wesonga H. O. (2016). Prevalence of contagious caprine pleuro-pneumonia in pastoral flocks of goats in the Rift Valley region of Kenya. *Tropical Animal Health and Production*.

[43] Ozdemir U., Ozdemir E., March J. B., Churchward C., Nicholas R. A. (2005). Contagious caprine pleuropneumonia in the Thrace region of Turkey. *Veterinary Record*.

[44] (APHRD) A., PHRD (2010). *Animal and Plant Health Regulatory Directorate, Version 1*.

[45] Wesonga H. O., Lindberg R., Litamoi J. K., Bölske G. (1998). Late Lesions of Experimental Contagious Caprine Pleuropneumonia Caused by Mycoplasma capricolum ssp. capripneumoniae. *Journal of Veterinary Medicine, Series B*.

[46] Wesonga H., Litamoi J., Kagumba M., Wakhusama E. (1993). Relationship between clinical signs and early lesions of contagious caprine pleuropneumonia caused by Mycoplasma strain F38. *Small Ruminant Research*.

[47] Matios L., Tesfaye S., Gelagay A., Eyob E., Gebremikael D., Tadele T. (2014). Seroprevalence of contagious caprine pleuropneumonia and field performance of inactivated vaccine in Borana pastoral area, southern Ethiopia. *African Journal of Microbiology Research*.

[48] Yousuf E., Melaku A., Bogale B. (2012). Seroprevalence of contagious caprinepleuropneumonia in Dire Dawa provisional administrative council, EasternEthiopia. *Journal of Veterinary Medicine and Animal Health*.

